# Local and landscape factors shape alpha and beta trophic interaction diversity in urban gardens

**DOI:** 10.1098/rspb.2023.2501

**Published:** 2024-05-22

**Authors:** Carlos Martínez-Núñez, Joan Casanelles Abella, David Frey, Andrea Zanetta, Marco Moretti

**Affiliations:** ^1^ Department of Ecology and Evolution, Estación Biológica de Doñana EBD (CSIC), Calle Avenida Américo Vespucio, 26, Sevilla 41092, Spain; ^2^ Swiss Federal Institute of Aquatic Science and Technology EAWAG, Ueberlandstrasse 133, Dübendorf, Switzerland; ^3^ Urban Productive Ecosystems, TUM School of Life Sciences, Hans Carl-von-Carlowitz-Platz 2, Feising 85354, Germany; ^4^ Biodiversity and Conservation Biology, Swiss Federal Research Institute WSL, Zürcherstrasse 111, Birmensdorf 8903, Switzerland

**Keywords:** antagonistic interactions, biodiversity, green areas, interaction networks, management intensity, urban ecology

## Abstract

Promoting urban green spaces is an effective strategy to increase biodiversity in cities. However, our understanding of how local and landscape factors influence trophic interactions in these urban contexts remains limited. Here, we sampled cavity-nesting bees and wasps and their natural enemies within 85 urban gardens in Zurich (Switzerland) to identify factors associated with the diversity and dissimilarity of antagonistic interactions in these communities. The proportions of built-up area and urban green area at small landscape scales (50 m radius), as well as the management intensity, sun exposure, plant richness and proportion of agricultural land at the landscape scale (250 m radius), were key drivers of interaction diversity. This increased interaction diversity resulted not only from the higher richness of host and natural enemy species, but also from species participating in more interactions. Furthermore, dissimilarity in community structure and interactions across gardens (beta-diversity) were primarily influenced by differences in built-up areas and urban green areas at the landscape scale, as well as by management intensity. Our study offers crucial insights for urban planning and conservation strategies, supporting sustainability goals by helping to understand the factors that shape insect communities and their trophic interactions in urban gardens.

## 1. Introduction

In the Anthropocene, the process of urbanization is a strong driver of biodiversity decline [1,2]. Yet, cities are increasingly reported to sustain certain levels of biodiversity [3,4], preserve rare and endangered species [5] and promote novel interactions [6]. This has led to a growing recognition of the importance of preserving urban biodiversity [7,8] and acknowledging cities as valuable assets for species conservation [5]. Thus, understanding how urban biodiversity is assembled is key to further developing biodiversity management in cities [9].

To better understand urban biodiversity, it is crucial to study not only diversity patterns but also ecological processes, such as trophic interactions. Numerous studies have been conducted to investigate the impact of social–ecological factors on diversity patterns in cities [10]. Increasing urban intensity has been identified as a major driver that can modify the structural properties of ecological communities, specifically, the way species interact within and across trophic levels [1,11]. These changes can serve as early indicators of a disruption in ecosystem functioning [12,13]. Such alterations in species interactions may ultimately impact ecosystem processes and functioning [11,14]. While studies have been conducted on the impact of urbanization on species interactions in mutualistic systems [15,16], there remains a significant knowledge gap regarding antagonistic interactions at the whole community level [9,11,12]. Therefore, studying the properties of community-wide antagonistic interaction networks could help in gaining novel, complementary insights into the functioning of urban ecosystems.

In this regard, cavity-nesting bees and wasps, together with their natural enemies, represent an excellent system in which to investigate changes in antagonistic interaction networks across ecological gradients [17]. Cavity-nesting bees and wasps (hosts hereafter) can be sampled using standardized trap nests, which enable the collection of information on the host community, the food resources of the hosts and the antagonistic species that parasitize their nests, making it possible to investigate multiple types of interaction networks [17,18]. Studying these networks can shed light on the impact of multiscale factors on an important ecosystem function, with implications for pest control and pollination services [19,20]. Furthermore, this system has proven useful for bioindication because it exhibits high specialization and vulnerability to perturbations such as resource scarcity [18,21,22], serving as an early indicator of ecosystem disruption caused by environmental changes [17,18,22].

Multiple factors, such as habitat amount (e.g. urban garden size or amount of green infrastructure), resource availability (e.g. plant and host diversity) or the degree of anthropic disturbance (e.g. management intensity), can affect the antagonistic interactions between cavity-nesting hosts and their natural enemies in urban habitats. Regarding habitat amount and resource availability, the amount of green area with rich nesting and feeding resources is an important factor for insects, including bees and wasps [23,24]. For instance, urban habitats might support a low diversity of host species because they provide only a small number of flowering plants and prey, which are, in addition, highly isolated, leading to altered diversity or changes in the structure of trophic interaction networks [20,25,26]. In addition, habitat complexity (i.e. using vegetation structure as a proxy) could be another important aspect to consider in urban habitats [25]. Moreover, sun exposure could be an important driver of ectothermic taxa [27]. Sun exposure in urban habitats is sometimes low because adjacent or nearby buildings can overshadow these areas, reducing their temperature [28]. This often-overlooked impact could be important for ectothermic animals, as sun availability has been shown to play a key role in structuring communities in urban areas [29]. Furthermore, owing to the relatively small size of urban gardens and the strong contrast with their surroundings, there is probably a size-dependent but overall strong edge effect, which makes these habitats vulnerable to the characteristics of the surrounding landscape [30]. In particular, the amount of built-up area, green spaces, agricultural land or hedges at different scales very likely influences the capacity of urban gardens to support diverse host–enemy communities and interactions among them [12]. Yet diversity is important, not only within gardens. Understanding what environmental factors affect beta-diversity across different urban gardens is key to understanding larger-scale diversity patterns in cities [31] but this has rarely been explored.

**Figure 1.**
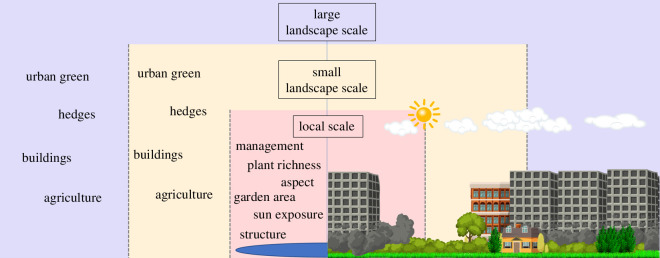
Environmental factors that might influence biodiversity in urban gardens at different spatial scales and different levels of environmental complexity. Multiple factors acting at multiple scales can affect biodiversity in urban gardens. We considered local-scale factors: garden size, management intensity, sun exposure, aspect (i.e. the direction that a topographic slope faces), plant richness and structural heterogeneity (measured with airborne laser scanning, ALS). We also considered the relative importance of small-scale (30 and 50 m radius) and large-scale (100, 250 and 500 m radius) landscape factors: agricultural area, built-up area, hedge area and urban green area.

Besides the aforementioned factors, urban biodiversity is also affected by other anthropogenic influences that are often challenging to measure. Among them, the intensity of management is often related to the number of resources and the habitat heterogeneity in a green area, and the number and quantity of agrochemicals used, which is also one of the main factors that might reduce biodiversity in these potentially biodiverse habitats [32]. However, owing to the small spatial scale of urban gardens and the decentralized nature of ownership and decision-making, assessing their management intensity has proven challenging. Therefore, despite the significance of this variable for improving biodiversity management in cities, its impact and relative importance in shaping the communities and interactions of cavity-nesting insects and their enemies remain unclear. Here, we investigated the relative importance of local and landscape factors in shaping the diversity and dissimilarity of antagonistic interactions between hosts and their natural enemies (i.e. parasitoids and kleptoparasites) in urban gardens across a wide gradient of urban densification and management intensity (figure 1). To this end, we used 255 trap nests with *ca* 233 cavities each to sample antagonistic interaction networks in 85 urban gardens (both home gardens and allotment gardens) in Zurich, Switzerland. We hypothesized that higher management intensity (more anthropogenic disturbance), lower sun exposure, a larger proportion of built-up area in the landscape and a smaller proportion of urban green area at both the local and the landscape scale would be the most important factors limiting the diversity of cavity-nesting bees and wasps in urban gardens and shaping the structure (i.e. dissimilarity across gardens) of these communities and their antagonistic interactions.

## 2. Material and methods

### (a) Study area and design

**Figure 2.**
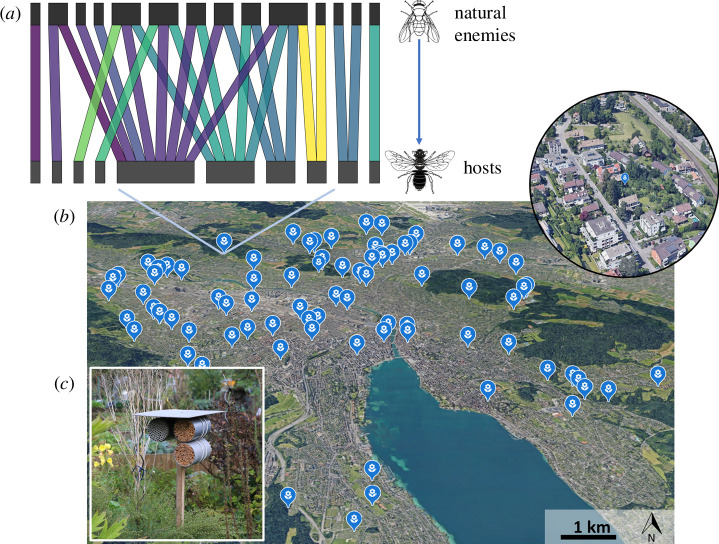
Study area and study system. A total of 255 trap nests (with *ca* 233 cavities each) were used in 85 private and allotment gardens located across a wide gradient of urbanization and management intensity. (***a***) An example of an interaction network between hosts and their natural enemies. Interaction colours are purely aesthetic, serving no informational purpose. (***b***) Locations of gardens sampled across an urbanization gradient in the city of Zurich, Switzerland (garden magnification shown in the top right corner). (***c***) Trap nests were used to sample cavity-nesting bees/wasps (hosts), their natural enemies and the antagonistic interactions among them. Interaction networks were built, and interaction diversity and dissimilarity were calculated. Trap nest picture courtesy of Marcus Schmidt. Satellite images retrieved from Google Earth.

We conducted the study in the city of Zurich, Switzerland (figure 2). Within the city of Zurich, we selected 85 urban gardens covering two wide, almost orthogonal, gradients of urban density and management intensity. Of the 85 gardens, 43 were allotment gardens (a piece of public land rented or used by people for growing vegetables and flowers) and 42 were domestic gardens (a piece of private land attached to a house and maintained by the owners for personal use, often for aesthetics and recreation). These gardens showed a similar interaction diversity (2.48 ± 0.46 mean ± s.d. in allotments and 2.21 ± 0.47 in private gardens) and were very similar in terms of management intensity (0.51 ± 0.09 in allotments and 0.51 ± 0.10 in private gardens). We selected 85 gardens by using a stratified sampling design to separate the effects of management intensity and the landscape-scale degree of urban densification. The selection was based on visual assessments of aerial images and field inspections. The independent strata were (i) a garden-scale qualitative management intensity and environmental heterogeneity gradient, which ranged from near-natural, wildlife-friendly garden designs to neat, production-oriented gardens; and (ii) a landscape-scale urbanization gradient. For garden selection, management intensity was initially qualitatively assessed and then quantified in detail by using questionnaires answered by garden owners (see “Management intensity and habitat structural heterogeneity” section). Using aerial images, we selected gardens within landscape sectors of varying proportional areas of impervious surface (i.e. built and paved) around each garden. To ensure independence among gardens, they were maximally spaced across the entire city to include all urban districts. The average area of the gardens was 312 ± 155 m^2^ (mean ± 1 s.d.). The average pairwise distance between gardens was 4.5 ± 2.2 km (mean ± 1 s.d.; min. 0.1 km, max. 11 km). This ensured, to a large extent, their independence regarding the focal species studied, since the foraging ranges of solitary bees are usually lower than these garden separation distances [33] (for more details about site study and site selection see electronic supplementary methods S1).

### (b) Sampling host–enemy interaction networks

To sample hosts, their natural enemies (i.e. parasitoids and kleptoparasites) and the interactions among them, we installed trap nests (also known as bee hotels) in the 85 gardens. This method is widely used to sample bees, wasps and their enemies across environmental gradients [17,19,21,34]. In February 2016, before the start of the flying period of the solitary bee and wasp hosts, we fixed three empty trap nests (~700 individual nests) on a wooden pole at a height of 1.5 m in a central and sunny place in each of the 85 gardens. The trap nest design followed that applied in other studies [17]. Specifically, the trap nests consisted of three pipes. The first two pipes contained 200–300 internodes of the common reed *Phragmites australis* (Cav.) and 5–10 bamboo internodes (figure 2). The reeds were 1–10 mm in diameter and 20 cm long to cover all the requirements of the cavity-nesting bee community. We filled the last pipe only with cardboard tubes of 7.5 mm diameter, which were specific to large-bodied bees and wasps (WAB Mauerbienenzucht; Konstanz, Germany).

After the end of the hosts’ flying period, in October 2016, we collected the trap nests and stored them at 6°C to simulate winter conditions. We subsequently opened all of the nests and recorded brood cells and natural enemies. Host abundance was quantified as the sum of brood cells per species in each garden, and enemy abundance was quantified as the sum of attacked brood cells per species in each garden. We transferred the nests containing brood cells into labelled test tubes, which we then sealed with cotton wool and transferred back to 6°C. In March 2017, we moved the test tubes to room-temperature storage, and we counted all emerging hosts and enemy individuals. Expert taxonomists identified them to the species or morphospecies level (see acknowledgements). With the information about the species present in each cavity, we built antagonistic interaction networks (see full metanetwork in electronic supplementary material, figure S1). We discarded 13 gardens from the analysis because we detected very few interactions to build interaction networks in them. These excluded gardens were very similar to those included in terms of the most important explanatory variables considered (electronic supplementary material, figure S2).

### (c) Garden characteristics

Using publicly accessible aerial photographs as templates (https://opendata.swiss/en/dataset?q=orthophotos), we manually mapped garden features, land-use types and the perimeter (excluding the house in private gardens) of each garden. We then digitized these features using ArcMap software (v. 10.3; ESRI, Redlands, CA, USA; https://www.arcgis.com/) to calculate the area (size) of each garden. We also calculated the aspect of the surface (i.e. the direction that a topographic slope faces) using a compass, and the mean number of hours of sun exposure a day during the summer months using a solar compass in the field (i.e. not based on GIS). Finally, in each garden, we conducted a complete inventory of all spontaneously growing and cultivated vascular plants by repeatedly visiting the gardens during the entire vegetation period (mean = 268 plant species, ranging from 151 to 402 species [35,36]).

### (d) Management intensity and habitat structural heterogeneity

Given that gardens are actively managed ecosystems, we devised a standardized, cumulative measure of management intensity. We constructed this measure based on the reported frequency of gardening practices by the garden owners and tenants, as detailed by Goddard *et al*. [37]. Specifically, we created this index using a Likert-style questionnaire consisting of 29 items (electronic supplementary material, table S1) that quantified the frequency of traditional gardening and horticultural practices on a 5-point scale [38]. We related the questionnaire items to five common land-use types: lawn, meadow, vegetable bed, flower bed and woody vegetation (electronic supplementary material, table S1). This index has proven useful in elucidating biodiversity patterns in other publications (e.g. [39]). In addition, we measured the structural heterogeneity in each garden by using sensitive remote-sensing techniques (i.e. LiDAR). This structural heterogeneity was estimated as the variability in vegetation height (s.d.) above 0.5 m (see electronic supplementary methods S1 for more details).

### (e) Landscape characterization

We used a multiscale characterization of the landscape surrounding each garden to examine the effect of landscape-scale change on arthropod communities in urban gardens. To do this, we used aerial photographs to characterize the land-use types surrounding each garden and digitized the maps using ArcMap software (v. 10.3; ESRI, https://www.arcgis.com/). Specifically, we considered four land-use types: building areas, urban green areas (excluding forests, agricultural areas and water bodies), agricultural areas and hedge-dominated areas. We then calculated the proportion of each focal land-use type in the surrounding area of the centre of each garden for multiple radii (i.e. 30, 50, 100, 250 and 500 m). We considered multiple spatial scalesbecause organisms perceive landscape-scale habitat features differently, depending on their activity ranges for routine movement. The 30 m radius scale (i.e. about 2800 m^2^) area captures the closest landscape variation, including the garden. Although most scales were highly correlated (especially those of contiguous radii; electronic supplementary material, figure S3), we explored all of them to find the scales at which changes in interaction diversity are better explained. For more details about all the predictor variables used to characterize the local- and landscape-scale environmental context (i.e. definition, scale, type, units and range), see electronic supplementary material, table S2.

### (f) Statistical analyses

We conducted all the analyses using R v. 4.0.4 [40], focusing on three main aspects.

First, to study the effects of the studied factors on the diversity of antagonistic interactions, we built quantitative interaction networks based on the frequency of co-occurrence between hosts and their natural enemies within the same cavity (co-occurrence at the cavity level implies parasitism between hosts and enemies in this system). Then, we calculated the Shannon diversity of interactions (interaction diversity hereafter) using the *bipartite* package v. 2.18 [41]. This index calculates the interaction diversity in a manner analogous to the Shannon diversity of species. It considers the number of different interactions instead of the number of different species, and the evenness (distribution of abundances) of these interactions. Then, we ran generalized additive models (GAMs) with interaction diversity as the response variable and each of the environmental factors as explanatory variables. As the main objective was to study the relative importance of each environmental factor, we ran one model for each factor. This made it possible to identify the unique contribution of each variable to the response variable without noise from other variables, while also minimizing multicollinearity issues. We then calculated the percentage of deviance explained by each model and the statistical significance of the explanatory variables from the model summary. In §3, we report the deviance explained by each model, the *F*-statistic of the smooth term, which is calculated differently from linear models, but shows the significance of including smooth terms in the model compared with an alternative null model, and the *p*‐value. We also show the effective and reference degrees of freedom that are specific to the fitting process of generalized additive models and inform about the degree of nonlinearity in smooth terms [42].

To explore potential interacting effects between variables at different scales, we also ran models testing for the interaction between landscape variables (i.e. built-up area and urban green area in 50 m around each garden) and several garden properties (i.e. sun exposure, garden size, plant richness, management intensity and structural heterogeneity), using a tensor product smooth (‘te’) in a generalized additive model.

Second, to gain more information on the mechanism promoting interaction diversity and to discern between the effects of environment on interaction diversity driven by species richness alone, we fitted three generalized additive models, using interaction diversity as the response variable and: (i) host richness, (ii) enemy richness or (iii) host plus enemy richness as the predictor variable. After evaluating the contribution and the effect of host and enemy richness separately (first and second models, respectively) on interaction diversity, we extracted the residuals from the third model and followed the same procedure used to examine the influence of different environmental factors on interaction diversity, but using the residuals as a response variable. Residuals of the third model represent the variability in interaction diversity not explained by the richness of hosts/enemies, allowing us to understand the effect of environmental variables on interaction diversity once the effect of host/enemy richness is removed [43].

Finally, to assess how differences in environmental conditions explained differences in the community structure of hosts, natural enemies and their interactions (i.e. occurrence and abundance of specific species/interactions) across the gardens, we calculated the Bray–Curtis distance between each pair of gardens and for each of the three response variables mentioned (i.e. hosts, natural enemies and interaction diversity). Community dissimilarity measures have been extensively used as an indicator of ecological distance [44]. Then, we calculated the Euclidean distance between each pair of gardens for each of the environmental factors. We used the Euclidean distance because it allows missing values and the comparison of geographical distance with environmental distances using the same type of measurement. We then ran generalized additive models to test for the relative importance of each environmental factor, following the same process as in the first analysis. Moreover, in order to identify the mechanism that contributed to variations in interaction diversity among gardens, we assessed beta-diversity in species interactions. This involved partitioning beta-diversity into differences owing to turnover and those owing to nestedness [45].

For all generalized additive models, we used a smoothing basis dimension of k = 3 (the same in all the models) to avoid overfitting and to favour ecological interpretability, and a shrinkage version of a cubic regression spline (bs = ‘cs’) (i.e. to penalize overly complex functions) to allow nonlinear responses. These parameters are commonly employed in ecology literature (e.g. [46]).

We ran the generalized additive models using the ‘gam’ function in the *mgcv* package *v. 1.8-36* [47]. We used the *ggplot2* package v. 3.3.6 to draw plots [48] and the *visreg* package v. 2.7.0 to calculate the effects [49]. Plots of the residuals showed that all the models met the assumptions of normality and independence of residuals.

## 3. Results

**Figure 3.**
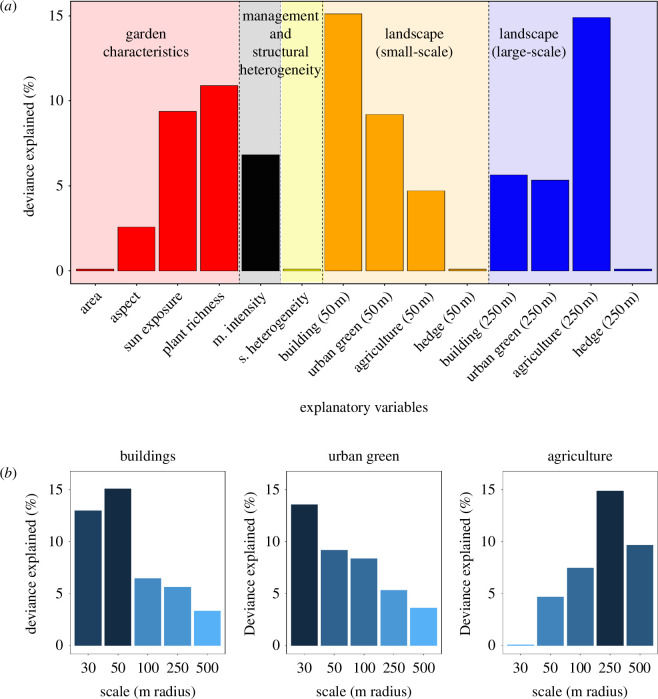
Relative importance of the different predictors to explain interaction diversity in host–enemy interaction networks. (***a***) Percentage of deviance in interaction diversity explained by each response variable. Factors are shown grouped into categories to help readability: garden characteristics, management (**M**) intensity and structural (**S**) heterogeneity, small-scale landscape composition (proportion of built-up area, urban green area, agricultural area and hedge area in a 30 or 50 m radius around each garden), and large-scale landscape composition (proportion of built-up area, urban green area, agricultural area and hedge area in a 100, 250 and 500 m radius around each garden). Blocks and colours show conceptually different types of explanatory variables. (***b***) The percentage of deviance explained by landscape variables at different scales. Bar colours represent the magnitude of the deviance explained.

Overall, we identified 46 host taxa (mean = 12 species, 1–22 species per garden) and 44 enemy taxa (mean = 7 species, 1–15 species per garden) across all the gardens (electronic supplementary material, tables S3 and S4). Of these, 77% of the hosts and 84% of the enemies were resolved to the species level. These animals were involved in ~4500 antagonistic interactions (178 different ones; mean = 11 interactions, ranging from 1 to 27 interactions per garden). At the local scale (garden), sun exposure (9% deviance explained, *F* = 2.94, effective degrees of freedom (edf) = 0.98, reference degrees of freedom (ref.df) = 2, *p*= 0.009), plant richness (11%, *F* = 3.72, edf = 1.83, ref.df = 2, *p* = 0.022) and management intensity (7%, *F* = 2.08, edf = 1.55, ref.df = 2, *p* = 0.076) were the three variables that best explained interaction diversity (figure 3*a*). At the small landscape scale (50 m radius), the amount of built-up area was the variable explaining the most deviance (15%, *F* = 4.57, edf = 1.30, ref.df = 2, *p* = 0.003), followed by the amount of urban green area (9%, *F* = 2.91, edf = 1.05, ref.df = 2, *p* = 0.012). At the large landscape scale (250 m radius), agricultural land was the most important variable determining interaction diversity (15%, *F* = 5.448, edf = 1.71, ref.df = 2, *p* = 0.003; figure 3*b*). In general, we found no meaningful interactions between landscape and garden variables (ΔAIC < 2 between models with and without interaction). However, we found a significant interaction between management intensity and urban green areas (50 m) (ΔAIC = 2.47, 19% deviance explained, *F* = 1.60, edf = 3.11, ref.df = 8, *p* = 0.004). High management intensity cancelled out the positive effects of urban green area on interaction diversity (electronic supplementary material, figure S4).

**Figure 4.**
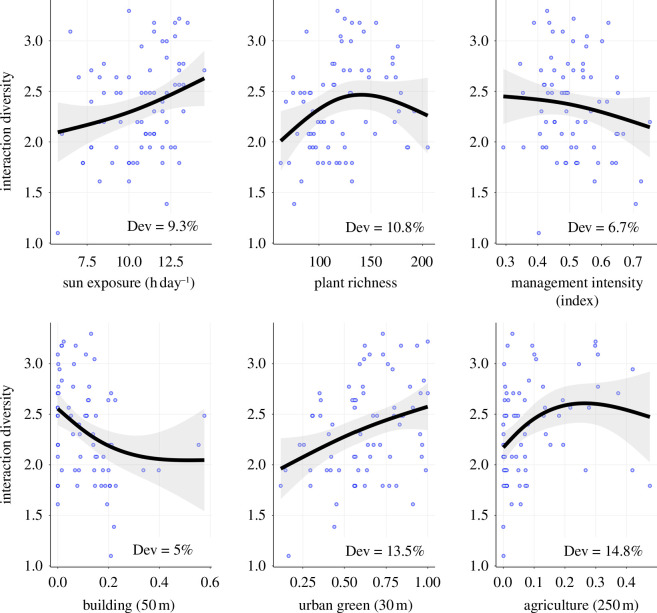
Effects of the most important predictor variables on host–enemy interaction diversity within urban gardens. The effects predicted by generalized additive models are shown (black lines). Shaded grey bands represent the 95% confidence intervals, and blue circles indicate the observed values, representing an urban garden each (*n* = 72). The distances in brackets refer to the radius (in metres) of the buffer where proportions of built-up area, urban green area and agricultural area were measured. Plant richness is expressed as the number of plant species. Dev = deviance explained.

The influence of the most important local factors on interaction diversity between hosts and enemies was found to be positive for sun exposure and plant richness, but negative for management intensity (figure 4). When examining the surrounding landscapes, the impact of built-up areas within 50 m was negative (stabilizing above 20% of built-up area; see figure 4), while the impacts of urban green areas and agriculture within 50 and 250 m, respectively, were positive (figure 4).

Host and enemy richness were important drivers of interaction diversity, explaining 86% of its variability (electronic supplementary material, figure S5). After removing the effect of host and enemy richness, sun exposure (positive effect), built-up area in the landscape (50 and 250 m; negative effect), green area in the landscape (50 m; positive effect) and agricultural area in the landscape (250 m; positive effect) were still the most important factors affecting interaction diversity in urban gardens (electronic supplementary material, figure S6).

**Figure 5.**
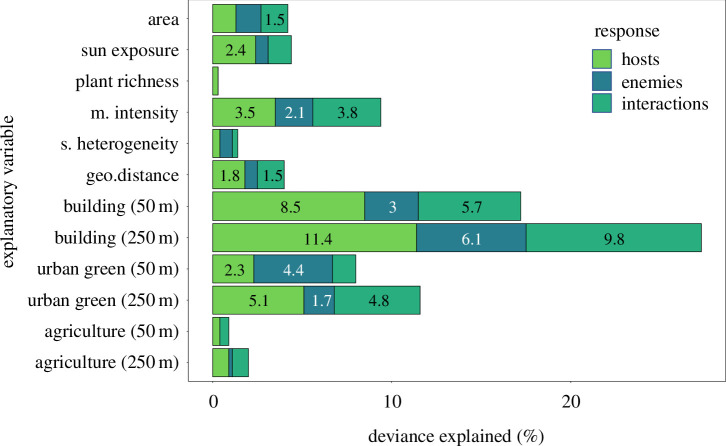
Contribution of environmental differences between gardens to explaining the dissimilarity in the community structure (i.e. occurrence and abundance) of hosts and enemies, and their interactions. Percentage of deviance in the garden pairwise dissimilarity (i.e. Euclidean distances) of the community structure of hosts, enemies, and their interactions, explained by pairwise differences in garden environmental factors. The deviance explained was estimated using generalized additive models. M. intensity, management intensity; geo. distance, geographic distance; s. heterogeneity, structural heterogeneity. Numbers show the percentage of deviance explained (for aesthetic purposes, these are omitted when they explain less than 1.5%).

**Figure 6.**
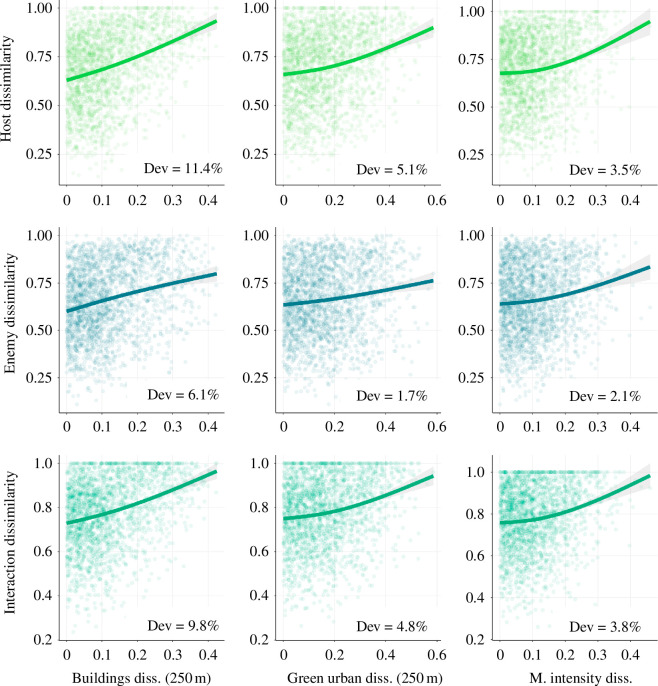
Effect of pairwise distances in garden landscape built-up area, urban green area and management intensity on the dissimilarity of hosts’/enemies’ community structure and their interactions. The effects predicted from generalized additive models are shown (straight lines), along with 95% confidence intervals (shaded bands) and observed values (dots, representing pairwise distances between gardens, *n* = 2314). Bottom axis labels: building diss. (250 m), dissimilarity in built-up area at the scale of 250 m radius around each garden; green urban diss., dissimilarity in urban green areas at the scale of 250 m radius around each garden; M. intensity diss, dissimilarity in garden management intensity. Dissimilarity was measured using the Bray–Curtis distance for communities/interactions and the Euclidean distance for environmental variables. Dev, deviance explained.

Differences in the structure of host communities, enemy communities and interaction diversity across gardens were best explained by differences in the built-up areas within 50 m and, to an even greater extent, within a 250 m radius around the gardens (figure 5). These differences accounted for 11.4% (*F* = 160, edf = 1.72, ref.df = 2, *p*< 0.001), 6.1% (*F* = 84, edf = 1.58, ref.df = 2, *p* < 0.001) and 9.8% (*F* = 135, edf = 1.75, ref.df = 2, *p* < 0.001) of the variation, respectively (figure 5 and electronic supplementary material, figure S5), with effects being highly significant, far more than the dissimilarity explained by geographic distance alone (electronic supplementary material, figure S7; hosts= 1.98% deviance explained, *F* = 24.55, edf = 1.96, ref.df = 2, *p* < 0.001; enemies = 0.8% deviance explained, *F* = 9.54, edf = 1.90, ref.df = 2, *p* < 0.001; interactions = 1.66% deviance explained, *F* = 20.42, edf = 1.95, ref.df = 2, *p* < 0.001). Differences in urban green areas, particularly at the 250 m scale, and differences in management intensity were also relatively good predictors of variations in hosts, enemies and their interactions, while the other potential predictors had substantially lower importance (figure 5). Differences in the three most important predictors (i.e. built-up area, urban green areas and management intensity) were positively linked to differences in host community, enemy community and their interactions (figure 6). Beta-diversity across gardens was overall very high (0.96) and was mainly owing to turnover (0.93) rather than nestedness (0.03).

## 4. Discussion

In this study, we investigated the relative importance of multiple local and landscape factors and their impact at different scales in shaping the alpha- and beta-diversity of trophic interactions between cavity-nesting bees and wasps and their natural enemies in urban gardens. Our results reveal that local management intensity, the amount of built-up area and the amount of urban green area at small landscape scales (30–50 m radius) are the three main factors explaining alpha and beta host–enemy interaction diversity across urban gardens in Zurich (Switzerland).

The observed negative effects of built-up area and the positive effect of green space area in the landscape surrounding urban gardens on antagonistic interaction diversity are in line with the habitat amount hypothesis [50]. This framework states that larger amounts of habitat patches, even if fragmented, can support biodiversity [51–53] and complex networks [54] across landscapes. This has important implications in cities, because most urban habitat patches are small [55]. These results complement findings from previous studies showing that landscape-scale factors can play an important role in determining the biodiversity of urban green spaces [56], and trophic interactions [57,58].

Interestingly, the promotion of trophic interaction diversity was mainly driven by an increased diversity of hosts and enemies in gardens under these conditions, which could be caused by bottom–up and top–down effects. In fact, whether food webs are resource- (bottom–up) or predation- (top–down) controlled has been a point of debate in ecology [59–61]. In our study, as in other contexts [61], both types of controls might be responsible for the observed patterns. On the one hand, bottom–up effects could boost interaction diversity by promoting higher trophic levels through increased availability of lower trophic levels such as host [62] and plants [60,63]. On the other hand, while top–down effects are reported only rarely, a higher diversity in high trophic levels could also favour host species richness by alleviating competitive pressure by dominant host species [59,64]. Both mechanisms can be affected by local and landscape factors in urban environments [65]. Overall, actions focused on increasing resources for consumers, such as habitat restoration and expansion [66], while supporting higher trophic levels too (i.e. enemies) are of utmost importance to better support urban biodiversity. Furthermore, the fact that the amount of habitat (i.e. built-up area and green area) at the landscape scale also moderated interaction diversity directly, regardless of enemy and host species richness, suggests that these landscape factors can also modify the composition of species, shaping interaction preferences and the potential interacting partners. This expectation was supported by the high turnover of species observed across sites and the evidence that differences in the amount of area covered by buildings and green spaces contributed significantly to differences in the structure of host communities, enemy communities and their interactions across urban gardens. This pattern might be widespread, since other authors have likewise shown that antagonistic interactions in a simplified herbivore–enemy model system vary in response to urbanization [26].

We found management intensity to be another important factor for the diversity of cavity-nesting bees and wasps and their natural enemies in urban gardens. Specifically, our results show that higher management intensity (i.e. higher frequency and intensity of interventions and the use of agrochemicals; see electronic supplementary material, table S1) is associated with a reduction in the interaction diversity between hosts and their natural enemies. This effect was so important that it reduced the benefits of a landscape with many green areas. This finding connects biodiversity loss and reduced ecosystem functioning owing to intensified urban garden management, as reported previously [37,38]. Therefore, along with increasing the diversity of plants, reducing the frequency and intensity of interventions in urban gardens can be an effective way to promote their quality and, as a consequence, biodiversity and ecosystem processes in cities [67].

In our study, the amount of sun exposure, and thus the temperature, in urban gardens were positively associated with diversity. Temperature is a major factor influencing the diversity of ectothermic taxa [27] across scales in urban (e.g. [29]) and non-urban ecosystems [68], as it regulates metabolism, activity time and consequently population- and community-level responses [68]. In urban areas, ongoing densification often results in an increase in building height and shadowing effects, which might result in negative consequences for biodiversity [69], yet this has been little studied.

Finally, some local factors, such as garden size and garden structural heterogeneity, were not significant predictors either of hosts, their natural enemies or their interaction diversity in urban gardens. These factors did not significantly influence the effects of other predictors in our study either. These results suggest that habitat quality, inferred, for instance, from plant richness or sun exposure, is more important than the size of the garden or its structural complexity for these communities and that the amount of habitat in the landscape might be more important than some local factors for these species [30]. In addition, these findings reinforce the idea that the available urban habitat is determined by a matrix of usually small patches, in line with what has been observed in other ecosystems [50,52]. Despite the general importance of these results, it is key to acknowledge that the study area is a specific city in a developed country, and the urbanization process has global implications with a wide heterogeneity of factors characterizing cities [70].

Our study points to future research directions. First, it is important to delve into how multitrophic relationships and different local and landscape factors affect individuals’ growth, survival, reproduction and ultimately fitness in urban environments. Second, studies could be conducted to explore whether other taxonomic groups and types of interactions respond similarly to environmental constraints in urban ecosystems and to examine the potential of urban gardens to support ecosystem services based on biodiversity [9].

## 5. Conclusion

Promoting urban biodiversity is an essential step to fulfilling conservation goals [9] and increasing environmental awareness, as outlined in frameworks like the Post-2020 Global Biodiversity Framework (https://www.cbd.int/). In this context, it is equally important to assess the impact of environmental factors and more direct human actions, such as management intensity.

Among the various types of urban green spaces, small-sized green areas rich in resources, such as urban gardens, exhibit a significant potential to serve as biodiversity refuges [4]. Indeed, the substantial diversity that we observed in terms of hosts (46 species), enemies (44 species) and antagonistic interactions (178 interactions) within our study reinforces the notion that urban gardens can play a pivotal role in conserving rich communities in urban environments.

Our study underscores the advantages of increasing habitat availability at the landscape level while simultaneously providing good sun exposure and food resources (i.e. plant richness) at the local scale. These actions are crucial for supporting the communities of cavity-nesting bees and wasps, their natural enemies and their interactions. Therefore, maintaining the ecological functioning of urban gardens requires the efforts of various stakeholders acting at different scales. Specifically, garden owners and landscape gardeners, through their actions and preferences, play a vital role, along with urban planners, in making decisions regarding urban densification and restoration to ensure the continued contribution of urban gardens to biodiversity conservation.

## Data Availability

The code and the data used in this study are available in the following ENVIDAT repository [71]. Electronic supplementary material is available online [72].
